# Financial sustainability and the incorporation of technologies in the Brazilian Unified Health System: advances, challenges, and perspectives

**DOI:** 10.1590/1516-3180.2025.1436.15102025

**Published:** 2025-12-01

**Authors:** Gabriela Favaro Faria, Paulo Manuel Pêgo-Fernandes

**Affiliations:** I Health Sciences, Escola de Enfermagem, Universidade de São Paulo (USP). Assistant Director, Discipline of Thoracic Surgery, Instituto do Coração (InCor), Hospital das Clínicas, Faculdade de Medicina, Universidade de São Paulo (HCFMUSP), São Paulo (SP), Brazil.; II Vice-Director, Faculdade de Medicina, Universidade de São Paulo (FMUSP), São Paulo (SP), Brazil; Professor, Departamento de Cardiopneumologia, Faculdade de Medicina, Universidade de São Paulo (FMUSP), São Paulo (SP), Brazil; Director, Departamento Científico, Associação Paulista de Medicina (APM), São Paulo (SP), Brazil.

 The incorporation of new health technologies represents one of the greatest advances in modern medicine, enabling more precise diagnosis, personalized treatment, and less invasive surgical procedures. However, this progress has brought significant challenges to the Brazilian Unified Health System (SUS), whose mission is to guarantee universal and comprehensive access while operating within budget constraints and social inequalities. 

 In 2022, Brazil allocated 9.6% of its gross domestic product (GDP) to health, a percentage considerably lower than that recorded in developed countries. In the same period, per capita health expenditure stood at approximately US$ 1,700, adjusted for purchasing power parity, which represents less than one-third of the Organisation for Economic Co-operation and Development (OECD) average, estimated at US$ 5,300. In contrast, nations such as the United States (US$ 12,740), Switzerland (US$ 8,910), Norway (US$ 8,640), and Germany (US$ 8,540) invested more than five times what Brazil did, highlighting the magnitude of the gap in national investments.^1 ,2^

 This underfunding scenario is especially challenging for highly complex areas such as oncology. Recent estimates indicate that per capita spending on cancer in Brazil is projected to rise by about 200% by 2050. This increase is driven by three main factors: population aging, which accounts for a projected growth of 124%; greater patient survival, which prolongs treatment duration by up to 47%; and the rising costs of new technologies and innovative therapies, such as high-cost medications and advanced diagnostic methods.^3^

 In recent years, the diagnosis and treatment of lung cancer have undergone significant transformations following advances in precision medicine. The management of the disease, once limited to general approaches, such as conventional chemotherapy and radiotherapy, has evolved into personalized strategies based on the molecular characteristics of the tumor and the clinical profile of each patient. Economic evaluation studies indicate that the incorporation of these technologies can increase per patient spending by up to four times compared with traditional chemotherapy regimens.^4^

 In the surgical field, the incorporation of minimally invasive techniques, such as robotassisted thoracic surgery has lead to greater precision, shorter recovery times, and a reduction in postoperative complications.^5,6^ This scientific and technological advancement, which represents a milestone in improving clinical outcomes, presents the challenge of balancing innovation with sustainability on SUS. The discrepancy between the actual costs of procedures and the amounts reimbursed by the public system highlights the structural fragility of its financing. 

 Pulmonary lobectomy, considered the gold standard for the treating of early-stage lung cancer, is currently reimbursed by SUS at only R$ 3,282, according to data from the Procedure Table Management System (SIGTAP).^7^ In contrast, a study comparing the costs of lobectomy performed by robotic-assisted thoracic surgery (RATS) and video-assisted thoracoscopic surgery (VATS) found very similar average costs between the two techniques, estimated at R$ 32,832.86 for RATS and R$ 32,500.00 for VATS, figures that exceed the amount currently reimbursed by SUS by almost tenfold.^8^

 Similarly, a study using the Society of Thoracic Surgeons (STS) database evaluated the hospital costs of lobectomy in patients with early-stage lung cancer. The results indicated an average cost of approximately US$ 45,000 per procedure, without complications.^9^

 This mismatch between the reimbursement offered by SUS and the real costs incurred by institutions compromises the sustainability of services, reinforcing the need for a periodic review of the reimbursement table and the adoption of more realistic costing methodologies that effectively reflect the complexity and inputs used in cancer treatments. 

 The debate regarding sustainability also extends to the incorporation of advanced surgical technologies such as robotic surgery. Introduced in Brazil in the mid-2000s and consolidated in private centers, robotic surgery has been applied in various areas, including gynecology, oncology, urology, and thoracic surgery. 

 Recently, SUS incorporated robotic surgery for the treatment of prostate cancer, following a recommendation by the National Commission for the Incorporation of Technologies into SUS (CONITEC), in a historic decision that opens the door to expanding its use to other specialties.^10^ Despite potential clinical benefits such as greater precision and faster recovery, its diffusion still faces significant barriers, especially due to the high cost of acquisition, maintenance, and disposable supplies. Furthermore, systematic reviews indicate that to date, there is no robust evidence of costeffectiveness when compared with established techniques such as video-assisted thoracic surgery.^11^

 The process of assessing the incorporation of technologies within SUS is one of the pillars of ensuring that decisions are made based on evidence. Coordinated by CONITEC, this process involves Health Technology Assessment (HTA), which examines not only the efficacy and safety of innovations but also their economic, social, and ethical impacts.^12,13^ An example of this rigor is the 2024 incorporation of new diagnostic tests for non-small cell lung cancer, such as positron emission tomography (PET) and RT-PCR for EGFR mutation, as well as the drug durvalumab for advanced stages.^14^ These technologies had been available in the private health sector since 2014,^15^ however, they were only recently incorporated into SUS after a detailed analysis of cost-effectiveness and budget impact. 

 On one hand, the incorporation of new technologies offers improvements in quality of care; on the other hand, the slow pace of the process and gaps in access generate a growing phenomenon: the judicialization of health. In 2024, the Ministry of Health recorded expenses of R$ 3.2 billion resulting from lawsuits related to medications, and over 50% of municipalities reported providing medications not incorporated into SUS through administrative channels.^16^ Scarcity of resources and social pressure intensify this scenario, creating distortions in financing and compromising equity. Recent regulations stating that non-incorporated medications should not be granted through lawsuits, except under specific circumstances, represent an important step toward rationalizing resources and reinforcing the legal security of the system.^17^

 Given this scenario, the central challenge is to balance innovation and sustainability to ensure that every Brazilian real invested yields proven clinical benefits and collective reach. Strategies such as the adoption of managed access models, risk-sharing agreements, periodic reviews of the SUS reimbursement table, enhancing efficiency, and strengthening the HTA are fundamental. The incorporation of value-based payment models has also emerged as a promising alternative, as it considers outcomes relevant to patients in relation to the total costs throughout the care cycle. 

 Financial sustainability, therefore, should not be seen as a barrier to innovation but as a foundation for ensuring that new technologies are cost-effective, accessible, and capable of transforming patients’ lives in an equitable and lasting way. Only then can the SUS continue to be a global reference in universal coverage, balancing the imperatives of technological modernization with the responsibility of maintaining public health that is both financially viable and socially just. 
